# Tensiones en una prótesis parcial fija unitaria implantosoportada en primer premolar inferior con diferentes materiales mediante elementos finitos

**DOI:** 10.21142/2523-2754-1101-2023-140

**Published:** 2023-03-26

**Authors:** A Mendez, H Coronado

**Affiliations:** 1 Carrera de Estomatología, Universidad Científica del Sur. Lima, Perú. anaismendez9@hotmail.com, hcoronado@cientifica.edu.pe Universidad Científica del Sur Carrera de Estomatología Universidad Científica del Sur Lima Peru anaismendez9@hotmail.com hcoronado@cientifica.edu.pe

**Keywords:** in silico, distribución de tensiones, corona de zirconio, corona de disilicato, corona metalporcelana, implante, in silico, stress distribution, zirconia crown, disilicate crown, metal-ceramic crown, implant

## Abstract

**Objetivo:**

Analizar las tensiones en prótesis parciales fijas unitarias implantosoportadas de metalporcelana, zirconio y disilicato de litio en primer premolar inferior a través del análisis de elementos finitos ante una fuerza de 500 N.

**Materiales y métodos:**

Se realizaron tres modelos de estudio, corona implantosoportada de metalporcelana, de disilicato de litio y de zirconio en primer premolar inferior; el implante dental fue de titanio grado V, basado en el modelo Bolt® de UniDentalDirect con conexión interna tipo ranurado (18 estrías) y una medida de 11,0 x 4,5 mm, pilar preformado y tornillo integrado. Los tres diseños tuvieron aplicación de fuerza vertical y oblicua (45°) de 500 N. Se realizó el modelado geométrico con el programa SolidWorks® 2017 y se obtuvieron resultados mediante el análisis de Von Mises a través del programa CosmoWorks®2017.

**Resultados:**

El menor valor de máxima tensión a nivel coronal ante fuerzas verticales y oblicuas se encontró en la corona de disilicato de litio, con 21,9 Mpa y 33,2 Mpa, lo que significa una diferencia mínima respecto de la corona de zirconio, que presentó 22,1 Mpa y 35,1 Mpa. A nivel del pilar, la corona de zirconio tuvo el menor valor de máxima tensión, con 18,6 Mpa y 28,1 Mpa. A nivel del tornillo, no hubo diferencias significativas.

**Conclusiones:**

Las coronas de metalporcelana, disilicato de litio y zirconio demostraron ser materiales de buena resistencia ante la compresión y tracción, pero en el diseño de la corona de zirconio se generó una tensión general más baja.

## Abstract (en)

**AIM:**

To analyze stress in a metal-ceramic, zirconia and lithium disilicate implant-supported unitary fixed partial prosthesis in the first lower premolar through finite element analysis at a 500 N force.

**MATERIALS AND METHODS:**

Three study models were carried out, metal-ceramic, lithium disilicate and zirconium implant-supported crowns in the first lower premolar. The dental implant was made of titanium grade 5 based on the Bolt® model of UniDentalDirect with internal grooved connection (18 grooves) and the implant had a size of 11,0 x 4,5 mm, preformed abutment and integrated screw. The three designs had vertical and oblique (45°) forces applications at 500 N. The geometric modeling was performed with the SolidWorks® 2017 program and the results were obtained through the Von mises analysis using the CosmoWorks®2017 program.

**RESULTS:**

The lowest value of maximum stress on crown level, under vertical and oblique forces, was found in the lithium disilicate crown with 21,9 MPa and 33,2 MPa, and with a minimum difference with the zirconium crown with 22,1 MPa and 35,1 MPa; on the abutment level, the zirconium crown had the lowest value of maximum stress with 18,6 MPa and 28,1 MPa; at the screw level, there were no significant differences.

**CONCLUSION:**

Metal-ceramic, lithium disilicate, and zirconia crowns proved to be materials of good compressive and tensile strength, but it was concluded that the zirconia crown design generated lower overall stress.

## INTRODUCCIÓN

La rehabilitación oral es una especialidad de la odontología que se dedica a la restauración integral del sistema estomatognático. Devuelve la armonía oral en función y estética. Los procedimientos van desde las operatorias simples hasta las complejas, incluida la rehabilitación oral con implantes dentales ^1^^,^^2^.

Sistemas libres de metal se vienen desarrollando en las restauraciones protésicas como las coronas de porcelana reforzadas con disilicato de litio o zirconio. Estos materiales presentan propiedades mecánicas, como el coeficiente de expansión térmica, semejantes a las del diente, así como biocompatibilidad biológica, resistencia al desgaste, estabilidad del color, entre otras, las cuales garantizan una adecuada transferencia de las tensiones masticatorias al sustrato remanente o los componentes protésicos ^3^^,^^4^.

La prótesis parcial fija implantosoportada beneficia la salud de los tejidos al ser mínimamente invasiva y presentar mejor funcionalidad, durabilidad y estética. Su alta tasa de éxito se debe a factores biomecánicos que son importantes para la supervivencia a largo plazo de las coronas sobre implantes ^5^^-^^8^. Un factor es la carga masticatoria emitida por fuerzas oclusales y oblicuas que aumentan la tensión sobre el tejido óseo y los componentes protésicos^.(^^7^^-^^11^. En la biomecánica de los implantes dentales se carece del ligamento periodontal, ante ello, las fuerzas oclusales y oblicuas son transmitidas por el implante y componentes periimplantarios, lo que provoca una reabsorción ósea ^12^.

Uno de los criterios de éxito de los implantes es la valoración de los cambios del nivel óseo crestal propuesto por Albrektsson *et al*. (1986). Este criterio es ampliamente utilizado en la actualidad y contempla una pérdida ósea marginal, después del primer año de carga funcional, de 1,5 mm durante el primer año y de 0,2 mm por año ^13^.

El método de elementos finitos (MEF) es utilizado en odontología para simular, determinar fuerza y tensión ^14^^-^^16^. Además, analiza el desempeño interno de estructuras e implantes, y evita las tensiones directas en el implante que podrían exceder el límite elástico y producir fallas estructurales ^17^^-^^21^.

Estudios previos como el de Bahadirli *et al*. ^22^ utilizaron MEF en modelos de corona de disilicato de litio con el implante de titanio y encontraron que el valor de tensión de Von Mises máximo fue de 40,8 MPa, en el modelo M2, y el mínimo de 20,8 MPa, en el modelo M8. La investigación de Della *et al*. ^23^ evaluó una corona de zirconio aplicando una carga de 200 N obteniendo la máxima tensión en zona oclusal interna. Ichim *et al*. ^24^ utilizó un modelo simulado en 3D de una pieza y aplicó fuerzas de 200 N sobre la vertiente externa a diferentes angulaciones. Demostró que una dirección de 20° generó bajos esfuerzos, mientras que los ángulos 30º y 40º mostraron mayor concentración a nivel cervicobucal.

Existe poca información sobre la distribución de tensión en coronas implantosoportadas en diferentes tipos de materiales. Conocer el análisis biomecánico de implantes ayuda a la planificación del tratamiento rehabilitador. Por ello, el propósito del presente estudio *in silico* fue analizar las tensiones en una prótesis parcial fija unitaria implantosoportada en corona de primer premolar inferior de tres materiales: metalporcelana, disilicato de litio y zirconio. La hipótesis nula por evaluar fue que no existe mayor tensión en la corona de disilicato de litio en comparación con las de zirconio y metalporcelana.

## MATERIALES Y MÉTODOS

El tipo de estudio de la presente investigación fue experimental *in silico*. El estudio fue aprobado por el Comité de Ética de la Universidad Científica del Sur (N° 304-CIEI-CIENTÍFICA-2021).

### Grupos de estudio

Los grupos de estudio se distribuyeron en tres grupos de modelos de coronas en primer premolar inferior implantosoportada (grupo 1: metalporcelana, grupo 2: disilicato de litio, y grupo 3: zirconia). La muestra tuvo como criterios de inclusión diseños realizados en el programa Solidworks® v. 2017, cuyas estructuras fueron desarrolladas con base en medidas establecidas, ensamblados en todas las simulaciones. Los diseños con errores en la construcción o incorrecto mallado fueron excluidos.

### Desarrollo de los modelos de estudio

El ensayo se realizó en laboratorio virtual a través de MEF. Tres modelos de estudio fueron conformados en un bloque de hueso tipo IV con 2 mm de grosor cortical (altura: 24,2 mm, ancho: 16,3 mm, y espesor: 16,3 mm) ^22^. Un implante dental con conexión interna tipo ranurado fue diseñado basado en el implante Bolt® de UniDentalDirect de 11,0 mm de longitud por 4,5 de diámetro, con su respectivo pilar con medida estándar (5,1 mm de altura) y un tornillo diseñado con aleación titanio grado V. El implante y el pilar se basaron en los gráficos y planos del fabricante (Bolt®). Una corona premolar inferior implantosoportada fue realizada en cada modelo siguiendo medidas estándares (altura: 8 mm, diámetro mesiodistal: 7 mm, y diámetro vestibulolingual: 8 mm) ^25^.

En el modelo 1 (control) se confeccionó una corona de porcelana (2 mm), una cofia metálica (0,5 mm) y un pilar personalizado. En el modelo 2 se realizó una corona de disilicato de litio (IPS e.max CAD, Ivoclar-Vivadent) de grosor 2 mm, una cofia del mismo material (0,5 mm) y un pilar personalizado. En el modelo 3, se hizo una corona de zirconio (IPS e.max ZirCAD, Ivoclar-Vivadent), una cofia del mismo material (0,5 mm) y un pilar personalizado. Todos los pilares tuvieron altura estándar de 5,1 mm.

Una simulación se realizó con implante intraóseo y osteointegrado, con coronas cementadas que dio un grosor de 0,4 mm de cemento resinoso, lo que dio como resultado un parámetro adecuado y un desarrollo correcto de mallado. Las propiedades mecánicas de los elementos se tomaron de los modelos numéricos obtenidos de los trabajos de Arinc *et al*. ^8^, Correa *et al*. ^26^ y Orozco *et al*. ^27^.

Las propiedades isotrópicas se obtuvieron para el hueso, titanio, metal, porcelana, etc. Los diseños tuvieron en cuenta las propiedades de los materiales como el módulo de elasticidad de Young (ɛ) y el coeficiente de Poisson (ν) para poder realizar la simulación de los resultados de las tensiones (análisis de Von Mises) de los modelos de estudios. 

El hueso cortical se valoró con 13,7 (ɛ) y 0,35 (ν); el hueso trabecular, con 1,37 (ɛ) y 0,3 (ν); y el titanio, con 110,0 (ɛ) y 0,35 (ν). Los materiales del implante y pilares del presente estudio fueron adecuados según las indicaciones. Además, los valores de las propiedades de las coronas de zirconio fueron incorporados con 269,0 (ɛ) y 0,25 (ν); en el caso del disilicato de litio, con 95,0 (ɛ) y 0,26 (ν). Por último, los materiales de la corona metalporcelana, con 97,0 (ɛ) y 0,33 (ν) para el metal y con 69,0 (ɛ) y 0,28(ν) para la porcelana ^26^^,^^27^.

### Aplicación de la dirección y fuerza a los modelos de estudio

**Figura 1:**
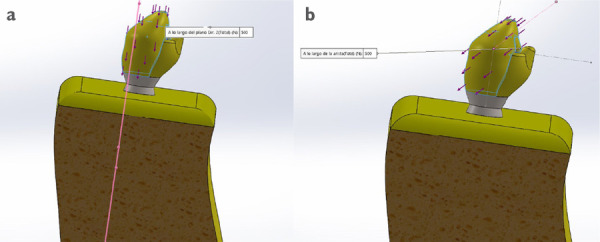
Dirección de la fuerzas. (a) vertical y (b) oblicua

La construcción de los modelos y la emisión de fuerzas se realizó con el programa SolidWorks® v. 2017. Una fuerza de 500 N fue aplicada en la cúspide vestibular en dos direcciones: vertical, en sentido al eje axial del diente, y oblicua (45°), en sentido al eje longitudinal del diente (Figura 1). Ambas direcciones de fuerza simularon las cargas generadas durante los movimientos masticatorios ^25^. Dos direcciones de fuerzas lateral también fueron consideradas: vertical y horizontal. Las cargas laterales simularon el movimiento de lateralidad ante masticación o al retornar a una oclusión habitual desde una posición lateral ^20^^,^^28^^-^^30^. Una fuerza oblicua (45°) fue seleccionada porque, según los estudios fotoelásticos, se confirma que, cuando las cúspides reciben una carga en dicha dirección, la concentración de estrés recae en el área cervical ^20^^,^^31^.

Los resultados y la determinación de máxima y mínima tensión se obtuvieron a través del análisis de Von Mises con el programa CosmoWorks® v. 2017. Este análisis permitió observar, a través de una escala progresiva de colores, la mínima y máxima tensión desde el color azul al rojo, respectivamente. Un profesional odontólogo, capacitado y certificado en el uso del programa SolidWorks y CosmoWorks® v. 2017 fue quien desarrolló los diseños.

## RESULTADOS

**Figura 2:**
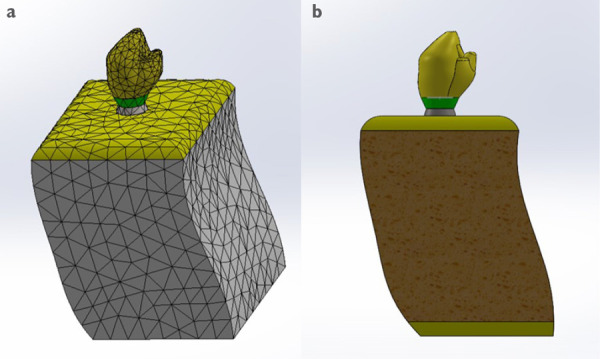
Modelo tridimensional. (a) Mallado y (b) Ensamblaje.

**Figura 3:**
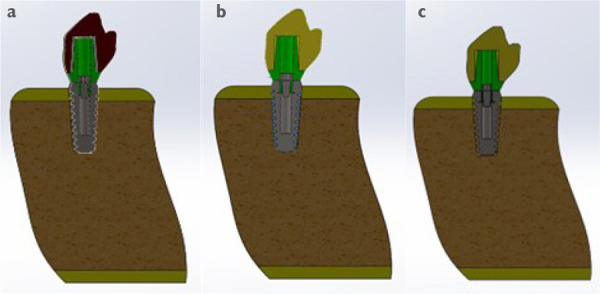
Ensamblaje del diseño de corona (a) metalporcelana, (b) disilicato de litio, y (c) zirconio.

**Figura 4:**
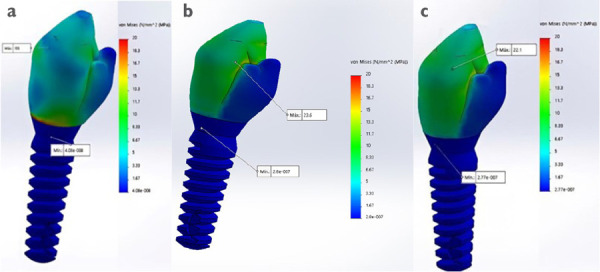
Diseño de la corona en CosmoWorks® en (a) metalporcelana, (b) disilicato de litio y (c) zirconio.

**Figura 5:**
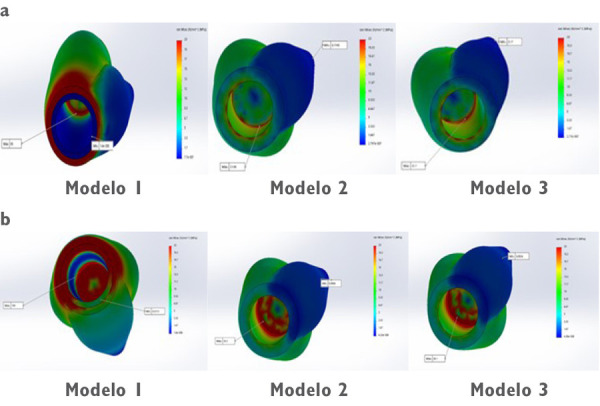
Análisis de Von Mises de las coronas con aplicación de 500 N en (a) fuerza vertical y (b) oblicua ante diseño de corona en modelo 1 (metalporcelana), modelo 2 (disilicato de litio) y modelo 3 (zirconio).

**Figura 6:**
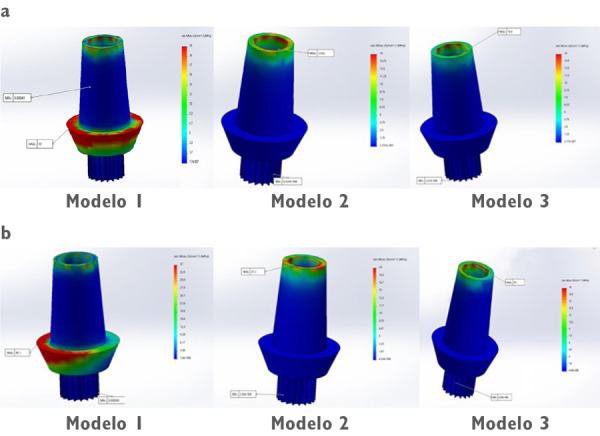
Análisis de Von Mises de los pilares con aplicación de 500 N en (a) fuerza vertical y (b) oblicua ante diseño de corona en modelo 1 (metalporcelana), modelo 2 (disilicato de litio) y modelo 3 (zirconio).

**Figura 7:**
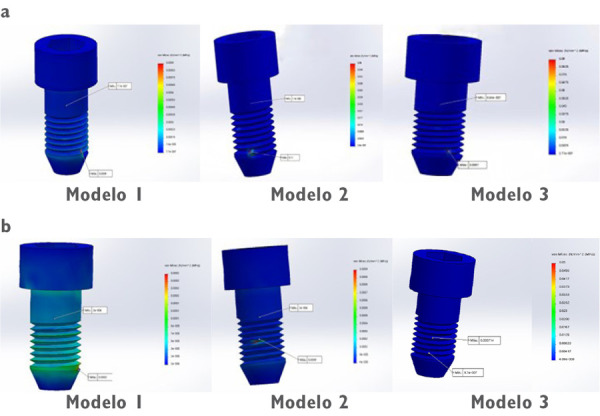
Análisis de Von Mises de los tornillos con aplicación de 500 N en (a) fuerza vertical y (b) oblicua. Las zonas más críticas están marcadas en rojo.

Un correcto enmallado se obtuvo a través del método de elementos finitos (Figura 2a), en el cual la convergencia de la malla generó los números de los nodos y elementos de cada modelo (Tabla 1). Los modelos tridimensionales fueron ensamblados según los parámetros de la literatura y metodología del estudio, y dieron como resultado los ensamblajes (Figura 2b) de las coronas de metalporcelana (Figura 3a), disilicato de litio (Figura 3b) y zirconio (Figura 3c). Los modelos fueron evaluados por el análisis de Von Mises con el programa CosmoWorks® v. 2017 y se obtuvo el diseño de las coronas de metalporcelana (Figura 4a), disilicato de litio (Figura 4b) y zirconio (Figura 4c). El análisis fue a nivel de corona (Figura 5), pilar (Figura 6) y tornillo (Figura 7).

**Tabla 1:** Números de los nodos y elementos de cada modelo de estudio.

	Modelo de metalporcelana	Modelo de disilicato de litio	Modelo de zirconio
Nodos	45225	36387	36387
Elementos	27573	21830	21830

En la Tabla 2 se analizó las tensiones máximas de los modelos de estudio ante fuerzas vertical y oblicua de 500 N. El mayor valor de máxima tensión coronal en ambas fuerzas lo obtuvo metalporcelana (85 Mpa en vertical y 155 Mpa en oblicuo) y se observaron valores menores en disilicato de litio (21,98 Mpa en vertical y 33,20 Mpa en oblicuo) y zirconio (22,10 Mpa en vertical y 35,10 Mpa en oblicuo), siendo similares en las dos últimas. 

**Tabla 2:** Máxima tensión de Von Mises de los modelos de estudio.

Tipos de modelos	Fuerza	Máxima tensión (Mpa)		
		Corona	Pilar	Tornillo
Metalporcelana	Vertical	85	82	0,00
	Oblicua	155	98,1	0,0002
Disilicato de litio	Vertical	21,98	23,62	0,11
	Oblicua	33,2	37,2	0,0009
Zirconio	Vertical	22,1	18,6	0,09
	Oblicua	35,1	28,1	0,0007

Modelo 1: Diseño de la corona metalporcelana. Modelo 2: Diseño de la corona de disilicato de litio. Modelo 3: Diseño de la corona de zirconio.

En la Figura 5 se visualizó una mayor tensión coronal en metalporcelana en la zona oclusal interna y cervical. Respecto de los pilares, hubo un comportamiento similar a las coronas con valores superiores en metalporcelana (82 Mpa en vertical y 155 Mpa en oblicuo), e inferiores en disilicato de litio (23,62 Mpa en vertical y 37,20 Mpa en horizontal) y zirconio (18,60 Mpa en vertical y 28,10 Mpa en horizontal), con diferencias entre estas dos últimas (Tabla 2). 

En la Figura 6 se observó que el mayor estrés de pilares estuvo en metalporcelana en cervical. En cuanto a la máxima tensión en el tornillo del modelo de metalporcelana en dirección vertical y oblicua fue 0, pero en disilicato de litio y zirconio en dirección vertical fue de 0,11 y 0,09 Mpa, respectivamente. En la Figura 7 se observó mayor estrés en la zona de las ranuras del tornillo en todos los modelos.

Según la Tabla 3, las mínimas tensiones de Von Mises de las coronas se aproximaron a 0 en el modelo de metalporcelana en dirección vertical y oblicua, y se alejó con disilicato de litio y zirconio en vertical (0,17 en ambas).

**Tabla 3:** Mínima tensión de Von Mises de los modelos de estudio.

Tipos de modelos	Fuerza	Mínima tensión (Mpa)		
		Corona	Pilar	Tornillo
Metalporcelana	Vertical	0,00	0,00	0,00
	Oblicua	0,02	0,00	0,00
Disilicato de litio	Vertical	0,17	0,00	0,00
	Oblicua	0,09	0,00	0,00
Zirconio	Vertical	0,17	0,00	0,00
	Oblicua	0,09	0,00	0,00

Modelo 1: Diseño de la corona metalporcelana. Modelo 2: Diseño de la corona de disilicato de litio. Modelo 3: Diseño de la corona de zirconio

## DISCUSIÓN

El objetivo del estudio fue analizar las tensiones en prótesis parciales fijas unitarias implantosoportadas de metalporcelana, zirconio y disilicato de litio en primer premolar inferior a través de MEF a una fuerza de 500 N en dirección vertical y oblicua (45°). La hipótesis fue corroborada de forma parcial hallando una menor tensión en la corona y pilares en los modelos de zirconio y disilicato de litio en comparación con los de metalporcelana.

En cuanto a los valores de estrés del modelo de corona, el estudio de Bahadirli *et al*. ^22^ desarrolló doce modelos de estudios enumerados del M1 a M12, con diferentes alturas de implantes y materiales de disilicato de litio como restauración. Se observó que el valor de mínima tensión de Von Mises en el modelo M8 fue de 20,8 Mpa y el valor de máxima tensión en el modelo M2 fue de 40,8 Mpa. Los resultados fueron similares a los encontrados en el presente estudio, donde el valor de mínima tensión de Von Mises fue de 21,9 Mpa y el valor de máxima tensión fue de 33,2 Mpa.

Con relación a las coronas, Della *et al* ^.(^^23^ realizaron un trabajo en MEF en prótesis fijas de tres piezas de zirconio y una corona de zirconio aplicando una fuerza de 200 N. Obtuvieron que la mayor tensión se encontró tanto en el área cervical como en pónticos y conectores para el puente fijo; mientras que el zirconio se obtuvo en zona interna oclusal. Dichos resultados fueron similares a los de este estudio, done la corona de zirconio tuvo la máxima tensión en la zona interna oclusal ante una fuerza de 500 N.

Una revisión sistemática previa mostró que las restauraciones libres de metal que tuvieron mejor desempeño clínico y funcional fueron las de zirconia y disilicato de litio por sistema CAD, seguidas por las restauraciones de recubrimiento con porcelana feldespática. Esto difiere de otros reportes que señalaron que el tiempo de vida de las restauraciones libres de metal y metalporcelana fue de 83,9 al 100% y de 92,3 al 95,5%, a los ocho años de seguimiento clínico, respectivamente. Al comparar los materiales, las coronas libres de metal mostraron una supervivencia clínica menor que las de metalporcelana; sin embargo, la biocompatibilidad de las restauraciones protésicas hacia los tejidos dentales debe ser un aspecto importante por considerar. El enfoque actual de la rehabilitación protésica va dirigida a aplicar tratamientos menos invasivos y con materiales biológicamente bioactivos ^32^. Ello es consistente con los resultados del presente estudio en el que las coronas metalporcelana, disilicato de litio y zirconio demostraron ser materiales de buena resistencia a la compresión y la tracción.

Estudios previos fueron consistentes con los resultados de este estudio. Benazzi *et al*. ^33^ estudiaron cinco tipos de cargas aplicadas a una primera premolar inferior a 100 N. Hallaron que la inclinación de la dirección de la carga generó una concentración del esfuerzo mayor. La cuarta (45°) y quinta (90°) carga tuvieron una distribución de esfuerzos similares, concentrándose las áreas de mayor estrés principalmente en la cúspide vestibular y extendiéndose hacia el tercio cervical. Palmara *et al*. ^34^ trabajaron con un modelo simulado en 3D de un premolar inferior ante una fuerza oclusal de 100 N con una dirección de 45° y otra vertical a lo largo del eje del diente. Observaron que la carga oblicua tuvo una mayor concentración de tensión. Ichim *et al*. ^24^ usaron un modelo simulado en 3D de un diente y aplicaron fuerzas de 200 N sobre la vertiente externa a diferentes angulaciones. Demostraron que una dirección de 20° generó bajos esfuerzos, mientras que a mayores ángulos (30° y 40°) se obtuvo mayor concentración de esfuerzos a nivel cervicobucal. En el presente estudio se brindó más alcances de la relación entre magnitud y dirección de la carga en un premolar inferior y su efecto en la distribución de esfuerzo a nivel cervical, y se obtuvo como resultado la mayor fuerza de tensión en el análisis a una dirección de fuerza oblicua de 45° y la concentración de estrés a nivel cervical y oclusal interno en las coronas de estudio.

La implicancia de utilizar el MEF fue obtener conocimiento clínico que confirma que las tensiones en los tipos de coronas en este estudio se distribuyeron de manera adecuada. La presente investigación utilizó un diseño experimental que resulta ser no invasivo y sí preciso para proporcionar datos cuantitativos. Sin embargo, existieron limitaciones para brindar datos cualitativos por tratarse de una simulación en estática y porque no brinda reacciones fisiológicas de los tejidos (músculo, hueso, etc.). Se recomienda realizar un estudio en MEF ante una fuerza mayor de 500 N para la simulación de parafunción o cargas oclusales fuertes en pacientes. Además, es necesario evaluar las tensiones considerando el implante y la estructura ósea, y en implantes asociados a una plataforma invertida ^35^^,^^36^.

## CONCLUSIONES

Se evidenció una mayor tensión en la corona metalporcelana, en comparación con las de zirconio y disilicato de litio. Por otro lado, el diseño de la corona de zirconio obtuvo mejores resultados en el análisis de tensiones de los pilares, al minimizar el estrés en las estructuras periimplantarias. Las tensiones en fuerzas oblicuas fueron más altas que las verticales.

## Fuente de financiamiento:

Autofinanciado.
